# Pathways to substance use: Examining conduct problems and parenting behaviors from preschool to adolescence

**DOI:** 10.1017/S0954579422001328

**Published:** 2023-02-06

**Authors:** Megan M. Hare, Elisa M. Trucco, Samuel W. Hawes, Michelle Villar, Robert A. Zucker

**Affiliations:** 1 Center for Children and Families, Department of Psychology, Florida International University, Miami, FL, USA; 2 Departments of Psychiatry and Psychology, University of Michigan, Ann Arbor, MI, USA

**Keywords:** antisocial behavior, conduct problems, parenting, substance use

## Abstract

While many studies have identified risk and protective factors of substance use (SU), few have assessed the reciprocal associations of child conduct problems (CP) and parenting practices and behaviors in the prediction of SU across development. A greater understanding of how these factors relate over time is needed to improve the timing of targeted prevention efforts. This study examined how child CP, parenting behaviors, and parents’ own antisocial behavior relate from preschool to adolescence and eventuate in SU. Participants included 706 youth (70.6% male; 89.7% white) enrolled in the Michigan Longitudinal Study. Data from waves 1 (ages 3–5), 2 (ages 6–8), 3 (ages 9–11), 4 (ages 12–14), and 5 (ages 15–17) were included. A random intercept cross-lagged panel model (RI-CLPM) examined reciprocal associations between parenting practices, parents’ antisocial behavior, and child CP over time (waves 1–4) and how these factors contribute to adolescent alcohol, cigarette, and marijuana use (wave 5). At the within-person level, negative parenting and parents’ own antisocial behavior had a strong influence in late childhood/early adolescence. Only child CP emerged as a significant predictor of SU. Results highlight the importance of early intervention and the potential influence of parenting and child factors throughout development in the prevention of SU.

Adolescent substance use (SU) can lead to a cascade of maladaptive outcomes, including academic failure, delinquency, suicidality, and SU disorders (Meier et al., 2012; Miotto et al., 2013; Schulte & Hser, 2013). This is concerning as 25.8% of high school seniors reported using alcohol, 19.5% reported using marijuana, and 4.1% reported smoking cigarettes in the past 30 days (Johnston et al., 2022). While research has identified multiple risk factors for adolescent SU, including child characteristics and parenting behaviors (Trucco, 2020; Trucco& Hartmann, 2021), limited work has examined how these factors unfold across development and how transactional influences increase risk or protection for subsequent SU. Understanding how these bidirectional associations relate over time and lead to adolescent SU can help identify potential critical periods and early targets for preventative efforts. The current study examines how child conduct problems (CP), parenting practices, and parents’ own antisocial behavior relate over time and eventuate in adolescent SU.

In the SU literature, many studies have focused on the cascade model of development, which posits that early risk factors (e.g. child externalizing behaviors) start a negative developmental trajectory that leads to the onset of SU (Dodge et al., 2008). It also highlights how disruptions in salient issues at each stage of development can predict negative outcomes at the next stage. Within this model, one of the strongest and most well-documented predictors of later SU is child CP (i.e., lying, stealing, getting into fights). This is important as children who display CP in early childhood tend to show a more persistent and chronic trajectory of antisocial behavior extending into later childhood and adulthood (Moffitt & Caspi, 2001), with some research suggesting these children go on to represent a large portion of adolescent crime (Offord et al., 1991). Furthermore, CP in early childhood has shown modest to moderate correlations with CP and more serious forms of antisocial behavior in middle childhood and adolescence (Caspi et al.,1995; Olson et al., 2000). Across development, children with CP may display behaviors such as physical aggression, lying, and destruction of property, while other behaviors (e.g., stealing) may appear as children get older and/or have the opportunity to engage in such behaviors. Given that CP can be identified as early as preschool, combined with findings that CP typically precedes SU initiation (Fergusson et al., 2007; Le Blanc & Loeber, 1998), it is important to understand not only the etiology of CP, but also how the early development of CP may impact later SU. Additionally, not all children with early CP initiate SU in adolescence (Campbell et al., 2000), underscoring the need to identify what factors (e.g. parenting) may contribute to CP and mitigate or exacerbate the risk for SU.

## Parenting

In addition to child factors, the cascade model also emphasizes the importance of socialization contexts, including parenting practices and behaviors (Dodge et al., 2008; Dodge & Pettit, 2003) in shaping an adolescent’s decision to engage in SU. Positive parenting (i.e. warm, supportive parenting) has been shown to serve as a protective factor by reducing the early onset of SU (Krohn et al., 2019; Tharp & Noonan, 2012; Zucker et al., 2008). When parents provide warm and supportive environments, youth are more likely to become self-confident, develop coping skills, and have increased resistance to deviant peers (Wills et al., 2003; Wolfradt et al., 2003; Vitaro et al., 2000), all of which protect against early SU (Mayberry et al., 2009). On the other hand, negative parenting (i.e., hostility, criticism) can lead to low levels of self-regulation in youth and increased engagement with deviant peers, leading to increased SU (Belsky et al., 2010; Hummel et al., 2013).

Parenting is also critical in the development and maintenance of CP. Though some children with CP can demonstrate moderate stability in symptoms over time, positive and negative parenting practices have been shown to impact the frequency and severity of CP across development. For example, interventions targeting positive parenting (e.g. positive reinforcement, warmth) have been shown to improve children’s early problem behavior (Dishion et al., 2008). In contrast, negative parenting and child behavior problems can follow an escalating coercive cycle (Dishion et al., 2006; Patterson, 1982). Parenting involves an ongoing dynamic process between the parent and child, such that negative parenting increases the problem behaviors of the child, which in turn, is followed by an increase in maladaptive parenting. Understanding how different parenting behaviors and child CP unfold across development and lead to adolescent SU is critical, as there tends to be decreases in positive parenting and increases in negative parenting during early adolescence (Kerr et al., 2012; Paikoff & Brooks-Gunn, 1991). As children with CP present unique challenges, parents may get frustrated over time and feel that their positive parenting behaviors have limited effects, leading them to decrease their positive behaviors and increase their negative, punitive parenting practices.

It is important to note that research supports homotypic continuity with respect to both positive and negative parenting practices. That is, positive and negative parenting practices are represented by the same behaviors and underlying process across development (e.g., Pauli et al., 2021; Smokowski et al., 2015). Further, a lack of positive parenting (e.g., low warmth) does not equate to high negative parenting (e.g., negative criticism) (Parke et al., 1994; Pettit et al., 1997). Numerous empirical investigations have shown dimensions of positive and negative parenting differentially relate to child outcomes (Karavasilis et al., 2003; Kawabata et al., 2011; Raby et al., 2015), even at the biological level (Hackman et al., 2018; O’Brien et al., 2021), which is why they are often differentially targeted in treatment (e.g., Eyberg, 1988).

Accordingly, examining the unique influence of positive and negative parenting behaviors together across development is critical to gain a more nuanced understanding on the influence of CP and development of adolescent SU.

Furthermore, consistent with Social Learning Theory (Bandura & Walters, 1977), parent modeling of antisocial behavior (e.g. aggression, impulsivity) may also lead to increased risk for CP and later SU in youth (Smith & Farrington, 2004; Thornberry, 2005). Although some research has demonstrated that parents with a history of antisocial behavior and young children report higher levels of stress, which in turn, may lead to ineffective parenting (e.g. negative parenting; Smith & Farrington, 2004), more work is needed in understanding how these dynamic associations influence each other over time. It may be the case that a more accurate depiction of the negative coercive cycle includes parent antisocial behavior, where parent antisocial behavior impacts CP both directly and indirectly (i.e. through increases in negative parenting), which in turn leads to increased risk for SU during adolescence. While both parenting practices and parent antisocial behavior have been linked to youth CP and SU, little work has examined these aspects of the parenting context in the same model to understand their joint role in the unfolding of youth problem behavior from early childhood to adolescence. Therefore, longitudinal research is necessary to model bidirectional effects to help illuminate critical periods where interventions aimed at reducing CP and SU can have the most utility.

## The current study

While there is supportive evidence for the cascade model of SU (Otten et al., 2019; Trucco et al., 2016), few studies have assessed the reciprocal associations proposed in the model in the prediction of SU. Furthermore, most of this literature has utilized a cross-lagged panel design (CLPM), which can conflate within-person and between-person effects, leading to potentially distorted estimates of cross-lagged effects (Berry & Willoughby, 2017; Hamaker et al., 2015). In addition, although research has linked CP to SU (Fergusson et al., 2007), and separately linked parenting to CP (Goulter et al., 2020; Pasalich et al., 2016) and SU (Cleveland et al., 2008; Kerr et al., 2012), few studies have concurrently examined these associations from early childhood to adolescence (Krohn et al., 2019; Sitnick et al., 2014). This substantially limits our knowledge of how CP, parenting practices, and antisocial behaviors become interwoven across the child’s development and contribute to early SU. A more precise understanding of how these factors relate over time is needed to identify critical developmental periods to inform more targeted prevention programs. Therefore, the current study fills these gaps by using a random intercept CLPM (RI-CLPM) to examine reciprocal associations between parenting practices, parent antisocial behavior, and child CP over time and how these factors contribute to alcohol, cigarette, and marijuana use (substances that are typically used among youth) during adolescence. Specifically, the RI-CLPM appropriately accounts for the trait-like, time-invariant stability of psychological constructs by parsing out the within-person and between-person effects across time. While between- and within person-effects will be presented, the current study focuses on the within-person associations, as they may help to identify modifiable targets for intervention (Berry & Willoughby, 2017; Hamaker et al., 2015).

Overall, we hypothesized a transactional relation in which parenting practices (i.e., positive and negative parenting) and child CP mutually influence each other over time. Regarding the predicted associations across the model, although research has shown the importance of positive parenting across development, previous research suggests that positive parenting may decline in late childhood/early adolescence, especially in children with CP (Kerr et al., 2012; Paikoff & Brooks-Gunn, 1991). Therefore, we predict that positive parenting will have a stronger association with CP in early/middle childhood (ages 3–8; waves 1–2) compared to later waves. On the other hand, as negative parenting has been shown to increase in late childhood/early adolescence (Laursen et al., 1998; Meeus, 2018), we hypothesized that negative parenting will have a stronger association with CP in late childhood/early adolescence (ages 9–14; waves 3–4) compared to earlier waves.

Given prior work demonstrating that a parent’s own antisocial behavior can increase children’s behavior problems (Smith & Farrington, 2004; Thornberry, 2005), we hypothesized that parent antisocial behavior will impact child CP across all waves (ages 3–14; waves 1–4). However, as previous work has shown that antisocial behavior in adulthood tends to be stable over time, with limited work suggesting child factors have a significant influence on parent antisocial behavior (Coie & Dodge, 1998; Conger et al., 2003), we hypothesized that child CP would not impact parent antisocial behavior at any wave. Lastly, given previous research demonstrating the unique importance of parent and child factors influencing SU (Trucco, 2020), we hypothesized that all parenting practices and parent antisocial behavior, as well as child CP, assessed during early adolescence (ages 12–14; wave 4) would uniquely be associated with use of all substances (i.e., alcohol, cigarette, and marijuana use) at wave 5 (ages 15–17).

## Materials and method

### Participants

Participants were youth enrolled in the Michigan Longitudinal Study (MLS), a prospective study examining the development of SU disorders among high-risk families (for a more detailed description of study design, recruitment strategies and participants, see Zucker et al., 2000). The study recruited community families in three risk categories: (1) high risk families had fathers convicted of drunk driving with a high blood alcohol concentration and an alcohol use disorder (24.9%); (2) moderate risk families were community-identified fathers with an alcohol use disorder (AUD) diagnosis, but no drunk driving offense (43.7%); and (3) control group/low risk families from the same neighborhoods but without an AUD diagnosis or a drunk driving conviction (31.4%). Mother diagnosis was free to vary in both high and moderate risk groups. Study exclusionary criteria ruled out the presence of fetal alcohol syndrome. The MLS maintained an 89% retention rate over the course of the study. Inclusion criteria of the original study protocol led to the sample being primarily male (70.6%), while the geographic region in which the study took place led to the sample being largely white (79.4%) (see Table 1 for full demographic information).

### Procedure

The study was approved by the Institutional Review Board at the University of Michigan. Parents and children completed assessments following initial enrollment (wave 1, ages 3 to 5; *M*_*age*_ = 4.40, *SD* = 1.01) with assessments occurring every 3 years. For this study, analyses were conducted on measures collected during wave 1, wave 2 (ages 6–8; *M*_*age*_ = 7.61, *SD* = 0.96), wave 3 (ages 9–11; *M*_*age*_ = 10.51, *SD* = 0.93), wave 4 (ages 12–14; *M*_*age*_ = 13.51, *SD* = 0.92) and wave 5 (ages 15–17; *M*_*age*_ = 16.58, *SD* = 0.97). Informed consent was obtained from the parents, and assent was obtained from the child after study procedures were reviewed. Given the proposed longitudinal models, analyses focused on participants with available data for at least two of the waves in the cross-lagged analyses (i.e., waves 1–4) for each variable (i.e., positive parenting, negative parenting, parent antisocial; *n* = 706; see Supplementary Table 1). Because of their generally stronger association to offspring during the childhood years, increased availability of complete data, and to reduce variablity in having multiple reporters, mothers were selected as the primary reporter for the measures. Participants included in the current analyses did not differ significantly from the full MLS sample (*N* = 1,250) on biological sex, race, income, parenting, or CP.

Participants with missing data at each time point were compared to participants with no missing data. Youth with available negative parenting wave 1 data (*χ*^2^ = 56.82, *p* < .001), positive parenting wave 1 data (*χ*^2^ = 56.82, *p* < .001), and child CP wave 1 data (*χ*^2^ = 22.53, *p* < .001), significantly differed by child sex. Namely, there was more missing information from parents of females compared to males. Additionally, youth with available negative parenting data at waves 1–4 (*χ*^2^ range 57.46–105.91, *p* < .001), positive parenting data at waves 1–4 (*χ*^2^ range 57.46–109.10, *p* < .001), parent antisocial behavior data at wave 1 (*χ*^2^ = 161.04, *p* < .001), child CP data at wave 1 (*χ*^2^ = 23.30, *p* < .001), and child CP data at wave 4 (*χ*^2^ = 31.78, *p* < .001) significantly differed by race. Namely, there was more missing information from parents of non-White youth. Differences in patterns of missingness across race and biological sex may be due in part to the original study design (see Zucker et al., 2000).

### Measures

#### Conduct problems

The Child Behavior Checklist (CBCL) (Achenbach & Edelbrock, 1991) was completed at waves 1–4 (ages 3–14). Mothers were asked to rate their child’s social and emotional functioning. This questionnaire has shown good construct validity and good test-retest reliability (Achenbach & Edelbrock, 1991). For the purposes of this study, a mean score of the delinquency subscale of the CBCL was used to assess CP. The delinquency subscale has been used in previous studies to represent CP in children, has shown to correlate with other measures of CP, and has differentiated children with and without conduct disorder (Ebesutani et al., 2010; Eiraldi et al., 2000). However, given that SU was an outcome of interest in the current study, the questions assessing SU were removed (*α* range = 0.66–0.80 across waves).

#### Positive and negative parenting

For waves 1 and 2, parenting was assessed via the Child Rearing Practices Report (Block, 1965). Mothers were asked to rate how descriptive statements are of their actual behavior toward their child. In line with previous research (Deković et al., 1991; Hofer et al., 2018; Rickel & Biasatti, 1982), two subscales were created: positive parenting (18 items) and negative parenting (21 items). For each subscale, scores were summed together and then an average was taken. Positive parenting broadly reflects warmth. Example items are, “I feel a child should be given comfort and understanding when upset,” and “I express affection by hugging, kissing, and holding my child” (*α*s = 0.66, 0.76). The negative parenting subscale broadly describes criticism and harsh parenting. Example items are, “I believe that scolding and criticism makes my child improve” and “I control my child by warning them about the bad things that can happen to them” (*α*s = 0.69, 0.75).

For waves 3 and 4, parenting was assessed using the Parent Perception Inventory (PPI-P) (Hazzard et al., 1983), an 18-item questionnaire developed to assess parenting styles from the parent’s point of view. The PPI-P is divided into two subscales: positive and negative parenting. Scores for each subscale were summed together and then an average was taken. Sample questions for positive parenting include, how often do you, “talk to your child when they feel bad,” and “help them to feel better, comfort them.” Sample questions for negative parenting are, how often do you, “tell your child they are no good, criticize them,” and “threaten them, tell them if they will get into trouble if they do something wrong.” The positive (*α*s = 0.78, 0.82) and negative parenting (*α*s = 0.78, 0.80) subscales demonstrated good internal consistency across waves. As the two parenting measures were on slightly different scales (i.e. 7-point Likert scale and 5-point Likert scale) scores for positive and negative parenting were *z*-scored on both measures across all waves.

#### Parent antisocial behavior

Mother’s own antisocial behavior was assessed using the Antisocial Behavior Checklist-R (ASB-R) (Zucker & Noll, 1980), where mothers were asked to rate their frequency of participation in a variety of antisocial activities. This instrument is a revision of an earlier antisocial behavior inventory utilized in the Rutgers Community Study (Zucker & Barron, 1973; Zucker & Fillmore, 1968), that has been modified so that items are also salient for adult antisocial activity. A total of 35 items were culled from the original adolescent measure and others were added from clinical descriptions of adult antisocial personalities. The ASB-R has demonstrated good reliability and validity and adequate test-retest reliability across populations (Eiden et al., 1999; Zucker & Noll, 1980), with the current study demonstrating good internal consistency (*αs* range = 0.80–0.84) across all waves. Example items include, “Shoplifted merchandise valued over $25” and “Been fired for poor job performance.” An average score was used.

#### Substance use

SU in adolescence was measured by the Drinking and Drug History Questionnaire - Youth Version (Zucker et al., 1990) assessed at wave 5. Alcohol use was assessed with a single item capturing the number of days the participant used per month during the past 6 months. Cigarette use was assessed with a single item, asking participants how frequently they have smoked cigarettes in the past 30 days (0 = not at all to 6 = two packs or more per day). Marijuana use was also assessed with a single item, asking participants how frequently they smoked marijuana in the past 30 days (0 = never to 9 = 500 times and above).

### Data analytic plan

First, zero-order correlations among the variables were examined. Then, a RI-CLPM was estimated in *MPlus* 8.1 (Muthén et al., 2017) using full information maximum likelihood to examine the bidirectional associations between child CP, parenting practices, and antisocial behavior on subsequent adolescent SU. Given that some variables were non-normally distributed, maximum likelihood parameter estimates with robust standard errors (MLR) was used to account for non-normality.

Autoregressive paths for each variable were systemically constrained and unconstrained and tested using the Satorra-Bentler Difference Tests to determine whether these paths should be freely estimated or constrained (Satorra & Bentler, 1988). The following fit statistics were used to evaluate model fit: chi-square (*p* > .05 excellent), comparative fit index (CFI; >.90 acceptable, >.95 excellent), root mean square error of approximation (RMSEA; <.08 acceptable; <.05 excellent), and the standard root mean square residual (SRMR; <.08 acceptable, <.05 excellent) (Hu & Bentler, 1999). The model included autoregressive and cross-lagged paths for positive parenting, negative parenting, parent antisocial behavior, and child CP across ages 3–14 (waves 1–4). Paths were also estimated between CP and each parenting variable at wave 4 on alcohol, marijuana, and cigarette use for ages 15–17 (wave 5).

### Post-hoc

Post hoc indirect effects of positive parenting, negative parenting, and parent antisocial behavior at wave 3 on SU at wave 5, through CP at wave 4 were also estimated. The Model Indirect command with 500 bootstrapped samples in Mplus was utilized to calculate a standardized indirect effect parameter and bias-corrected bootstrap confidence intervals. Importantly, it is not possible to estimate bias-corrected bootstrap confidence intervals while utilizing MLR. Therefore, indirect effects using the IND command and MLR estimation were compared. As results were the same, results for post hoc analyses are presented using bias-corrected bootstrap confidence intervals.

## Results

Means, standard deviations, and correlations for study variables are presented in Table 2. Utilizing the Satorra-Bentler Scaled Difference Tests, constraining the autoregressive paths to be equal across waves for positive parenting (Δ*χ*^2^ (2) = 22.71 *p* < .001), negative parenting (Δ*χ*^2^ (2) = 38.89, *p* < .001), parent antisocial behavior (Δ*χ*^2^ (2) = 45.78, *p* < .001), and CP (Δ*χ*^2^ (2) = .98, *p* = .615), did not result in a significant decrement in model fit, Therefore, all paths were constrained to be equal. The final model demonstrated acceptable model fit (*χ*^2^ (166) = 327.71, *p* < .001, CFI = .930, RMSEA = .050, 90% CI [.042, .057], and SRMR = .052).

### Model results

#### Between effects

Between-person effects for positive parenting were negatively correlated with between-person effects for negative parenting, CP, and parent antisocial behavior (see Table 3). Between-person effects for negative parenting were positively correlated with between-person effects for CP and parent antisocial behavior. Lastly, between-person effects for CP were positively correlated with between-person effects for parent antisocial behavior.

#### Within-person effects

**Figure 1.**
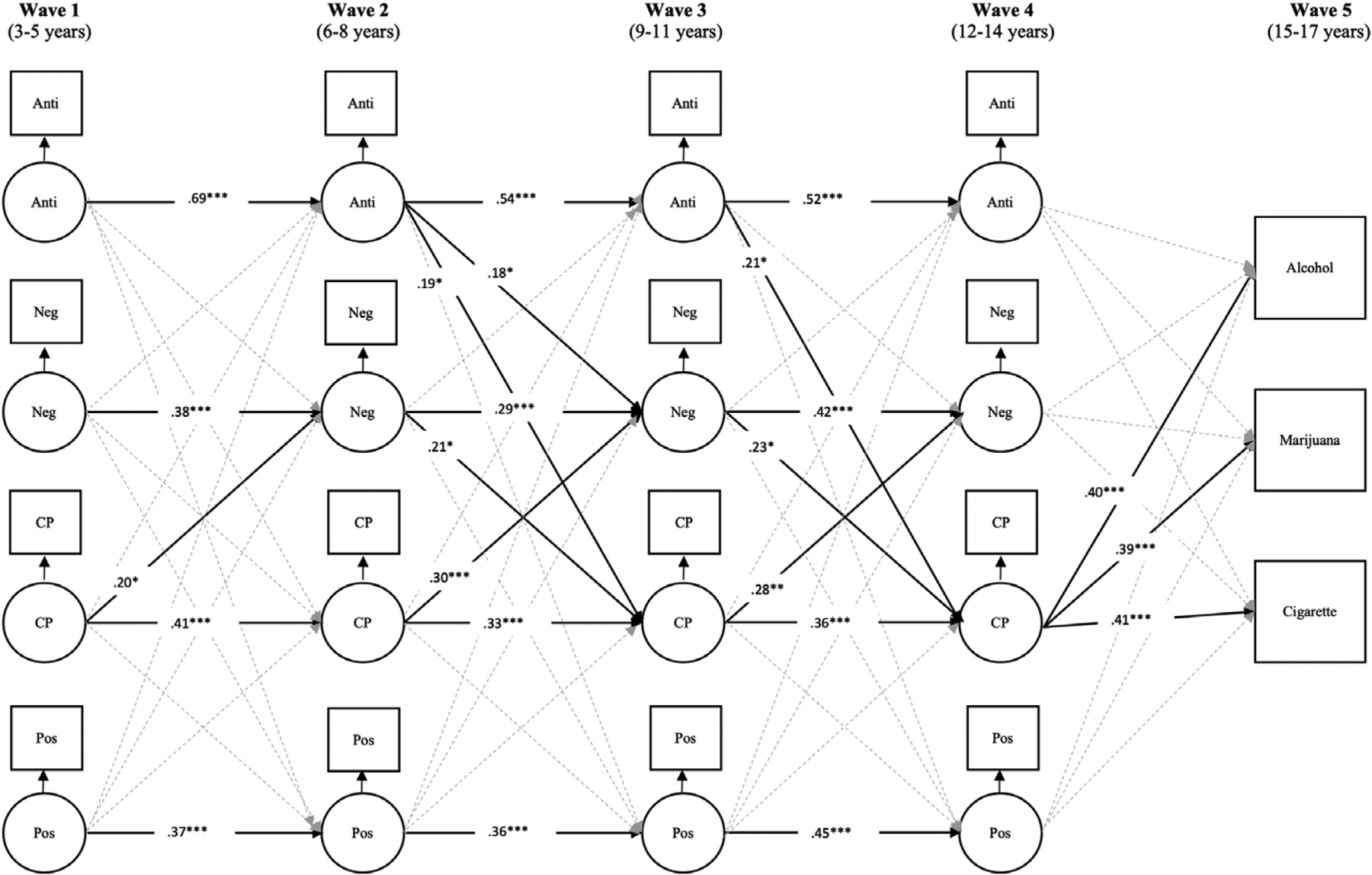
Bidirectional associations of positive parenting, negative parenting, parent antisocial behavior, and child conduct problems in the prediction of substance use. Figure shows significant associations. Within-time correlations were estimated but not shown in the figure for parsimony. Between-person effects are also not presented in figure (see Table 3). *p< .05, **p<.01, ***p<.001. Anti = parent antisocial behavior; Neg = negative parenting; Pos = positive parenting; CP = conduct problems.

CP at wave 1 were significantly associated with negative parenting at wave 2, such that more CP led to greater negative parenting (see Figure 1). There were no other significant cross-lagged paths from wave 1 to wave 2 (see Table 3).

Parent antisocial behavior at waves 2 and 3 were significantly associated with greater CP at waves 3 and 4, respectively. Parent antisocial behavior and CP at wave 2 were significantly associated with greater negative parenting at wave 3, which in turn lead to greater CP at wave 4. Additionally, CP at wave 3 was significantly associated with greater negative parenting at wave 4. Lastly, CP at wave 4 was significantly associated with greater alcohol use, marijuana use, and cigarette use at wave 5. Positive parenting, negative parenting, and parent antisocial behavior at wave 4 did not significantly predict SU at wave 5 (see Table 3).

### Post-hoc results

Negative parenting at wave 3 demonstrated a significant indirect effect on alcohol use (*β* = .05, *SE* = .02, *p* = .008, 95% CI [.01, .09]), marijuana use (*β* = .06, *SE* = .02, *p* = .008, 95% CI [.01, .08]), and cigarette use (*β* = .05, *SE* = .02, *p* = .009, 95% CI [.01, .08]) at wave 5, through child CP at wave 4. Similarly, parent antisocial behavior at wave 3 also demonstrated a significant indirect effect on alcohol use (*β* = .07, *SE* = .02, *p* = .002, 95% CI [.02, .11]), marijuana use (*β* = .07, *SE* = .02, *p* = .002, 95% CI [.01, .10]), and cigarette use (*β* = .06, *SE* = .02, *p* = .002, 95% CI [.02, .09]) at wave 5, through child CP at wave 4. In contrast, positive parenting at wave 3 did not demonstrate a significant indirect effect on alcohol use (*β* = −.02, *SE* = .11, *p* = .223, 95% CI [–.06, .01]), marijuana use (*β* = −.02, *SE* = .05, *p* = .224, 95% CI [–.06, .01]), or cigarette use (*β* = −.02, *SE* = .05, *p* = .224, 95% CI [–.06, .01]) at wave 5, through child CP at wave 4.

## Discussion

Despite numerous prevention and intervention efforts, SU remains an international public health problem. Preventing the onset of SU and/or delaying the age of SU initiation can significantly reduce the risk of later SU addiction severity (Harerimana et al., 2022). When examining bidirectional associations of parenting behaviors and child CP over time, multiple pathways to SU emerged. In line with cascade models (Dodge et al., 2008), child CP emerged as an early risk factor for adolescent SU. Inconsistent with our hypotheses, positive parenting showed no cross-lagged effects across development. Lastly, only CP was associated with later SU in adolescence.

The current study supports the cascade model utilizing more rigorous techniques (i.e., RI-CLPM), with greater CP at wave 1 leading to increased negative parenting at wave 2. On the other hand, parent antisocial behavior at wave 1 did not predict child CP at wave 2; however, parent antisocial behavior did predict CP at all subsequent waves. In line with Social Information Processing theory (Crick & Dodge, 1994; Dodge et al., 1986), young children may not be fully aware of their parents’ antisocial behavior. For example, children (ages 6–8) may be less aware if their parents shoplifted or why they lost their job. When children are this young, parents may also make more of an effort to hide these types of behaviors. Further, younger children may not have the means to engage in antisocial behaviors, and therefore only model antisocial behaviors when they are older and spend more unsupervised time with peers.

Although parent antisocial behavior and negative parenting at wave 1 had no significant cross-lagged effects to wave 2, as children moved into middle childhood to early adolescence (i.e., waves 2–4), coercive cycles between parent antisocial behavior, negative parenting, and CP emerged. For youth, the developmental period of late childhood/early adolescence, is marked by an increase in challenging rules and authority figures, independence from parents, peer influence, and exposure to more opportunities for risky behavior (Steinberg & Silk, 2002). This increase in autonomy, mobility, and inclination to challenge rules can lead to increased parent–child conflict. Parents may want more control and close-ness with their teen, while teens may get annoyed and want more independence, leading parents to become upset and adopt a more negative parenting style (Laursen & Collins, 2009; Smetana, 2011; Steinberg & Silk, 2002). In many families, this period of conflict is gradually resolved when youth get older, as parents come to respect the autonomy and decision making of their maturing child, while youth learn to communicate and regulate their emotions more effectively (Steinberg, 2001; Steinberg & Silk, 2002).

However, this negative coercive cycle may be more severe in youth with CP, as they tend to be more impulsive, display lower empathy, and affiliate with more deviant peers. As parents of youth with CP experience higher rates of parenting stress and more conflict in the parent-child relationship (Muñoz-Silva et al., 2017; Raudino et al., 2012), this developmental transition may be more difficult and more of a trigger for parents who also demonstrate antisocial behaviors. The current study highlights the importance of considering how parents’ own psychopathology, specifically antisocial behavior, may influence their own parenting practices in addition to child CP. Further, although no cross-lagged paths emerged from parent antisocial behavior and negative parenting at wave 1 to wave 2, both constructs play an important role across development. Therefore, it may be the case that early intervention on such risk factors may help prevent or reduce symptoms of CP, and hopefully, later SU.

In contrast with our hypotheses, positive parenting had no significant effect across any of the cross-lagged associations. Children high in CP, even at younger ages (i.e., prior to wave 1), may exhaust parents (Caprara et al., 2007; McLoyd, 1990), leading to lower levels of positive parenting (e.g., less warmth). However, as parents become more frustrated and feel that their positive parenting behaviors have minimal impact on shaping child behavior, lower levels of positive parenting may develop into increased levels of negative parenting (e.g., increased hostility). Results from between-person effects support this theory, indicating that parents with lower positive parenting across measurement waves reported higher negative parenting across measurement waves compared to parents high in positive parenting. From a prevention/treatment framework, it may be the case that these families, especially those at higher risk due to parental antisocial behavior or early CP, require early intervention. For example, programs aimed to prevent adolescent problem behaviors have been initiated beginning as early as the prenatal period (Olds, 2002), during infancy (Van Zeijl et al., 2006), and through the preschool period (Eyberg & Bussing, 2011; Webster-Stratton & Reid, 2018). While some of these programs have also demonstrated positive outcomes on child CP, they also focus on general indices of positive adjustment, maternal mental and physical health, and improving the mother–child relationship (Shaw, 2013). Further, at the between-person level, results indicate that across time, parenting and CP are related. Results suggest that parents with lower positive parenting are more likely to have greater negative parenting, CP, and antisocial behavior compared with parents high in positive parenting. Results suggest that these variables influence each other and may need to be considered as part of an overall intervention.

Lastly, although parenting behaviors and CP influenced each other across development, at wave 4 (ages 12–14) only CP emerged as having a significant direct effect on SU, further underscoring the importance of early intervention. While our findings are not in line with previous research showing parenting behaviors directly contribute to adolescent SU (e.g., Krohn et al., 2019), it is important to note that many of these models did not concurrently include a proximal measure of child CP or parent antisocial behavior. This highlights the importance of considering multiple parent and child factors over time to better understand early pathways to SU. Further, much of the past literature has not separated within- and between-person effects. It may be the case that some of the autoregressive and cross-lagged parameters in previous literature may represent an overestimation (Mulder & Hamaker, 2021).

Additionally, post-hoc analyses demonstrated that negative parenting and parent antisocial behavior at wave 3 had an indirect effect on SU at wave 5 through CP at wave 4. As youth have increased independence during adolescence, the direct impact of parenting behaviors on a youth’s decision to engage in SU may be reduced. Although parents are the key socialization unit during childhood and early adolescence, as more time is spent outside of the family, this shifts to peers becoming the primary socialization unit during middle to late adolescence (Catalano & Hawkins, 1996; Furman & Buhrmester, 1992). Therefore, it may be the case that other factors that have more direct links with deviant peer affiliation, such as parental monitoring, or peer relationships themselves may have a stronger, direct impact on SU and may be more important to target in treatment during this developmental period (Crawford & Novak, 2002; Rusby et al., 2018).

### Limitations

While this is one of the first studies to examine the bidirectional associations between parenting practices, parent antisocial behavior, and youth CP in the prediction of SU across development using a RI-CLPM, it is not without its limitations. The sample was predominantly white and male, limiting generalizability. Testing sex as a potential moderator in this study was not possible given the low percentage of females in the sample. As there are higher rates of CP and SU in males, and potential sex differences among parenting practices (Camacho-Thompson & Simpkins, 2020; Griffin et al., 2000), future work should examine whether sex moderates these associations. In addition, it may also be the case that mother’s own psychopathology or parenting style might influence their response in terms of reporting their own antisocial behavior and parenting practices, as well as the behavior of their child (i.e., CP). Future research should replicate these findings using more objective measures of parenting behaviors and child CP across multiple reporters (e.g., observation).

Additionally, the link between parent antisocial behavior and CP has been shown to have genetic underpinnings (e.g., Beaver et al., 2007), which is not accounted for in the current study. However, research has also demonstrated that non-biologically related caregiver’s antisocial behavior impacts child CP (e.g., Kerr et al., 2013), suggesting the influence of parents own antisocial behavior on child CP may also occur through socialization consistent with Social Learning Theory (Bandura & Walters, 1977). As this study employed secondary data analysis, it was also limited in terms of measurement, leading to separate parenting measures for waves 1–2 compared to waves 3–4. However, both measures assessed the same constructs (i.e., positive and negative parenting), had almost identical items, and were significantly correlated with one another across waves (*p*s <.05). While that does caution the interpretation of the cross-lagged paths that emerged across wave 2 to wave 3 for positive and negative parenting, similar cross-lagged paths were found across waves 2–3 (i.e., different parenting measures), as waves 3–4 (i.e., same parenting measure). Lastly, adolescent SU during the timeframe the study was conducted (1985–2007) may not represent current trends, necessitating replication.

## Conclusion

Overall, findings support previous research identifying CP as the start of the developmental cascade to SU, while adding that negative parenting and parents’ antisocial behavior should also be considered. Further, while focusing on parenting behaviors may indirectly impact SU once children reach early adolescence (i.e., 12–14), interventions should also focus on treating CP and reducing the risk for SU. Given previous findings that parents and peers influence adolescent SU (Elkington et al., 2011; Trucco, 2020), targeting various socialization influences in treatment could have utility (e.g., Multisystemic Therapy; Randall et al., 2018). Overall, our results highlight the importance of early intervention and considering different parenting and child factors throughout development in the prevention of SU, as different associations emerge across developmental periods.

## Supplementary Material

1

## Figures and Tables

**Table 1.** Demographics

	Total sample (*N* = 706)	Low risk (*n* = 222)	Medium/high risk (*n* = 484)
Family income (%)			
Under $4,000	1.6	0.0	1.4
$4,000-$7,000	3.3	0.0	4.8
$7,001-$10,00	3.0	1.4	3.7
$10,001-$13,000	3.5	1.0	4.8
$13,001-$16,000	3.8	3.3	4.1
$16,001-$20,000	8.5	9.0	8.5
$20,001-$30,000	17.3	8.1	21.5
$30,001-$50,000	36.7	51.9	29.8
$50,001-$75,000	15.3	19.5	14.6
over $75,000	3.4	1.9	3.1
Did not report	3.6	3.8	3.7
Parent degree earned (%)			
None	65.2	63.9	69.6
Bachelors	17.9	19.0	13.3
MA/MS	2.8	2.4	3.1
PhD, MD, DVM	0.4	1.0	0.2
Technical, vocational, AA	10.2	11.0	9.9
Did not report	3.5	2.9	3.9
Marital status (%)			
Never married	4.5	0.0	6.6
Currently married	87.3	93.3	8.5
Divorced/widowed	2.1	1.4	2.5
Did not report	6.1	5.2	5.6
Child sex (% male)^a^	70.6	69.0	71.9
Child race/ethnicity (%)			
White	89.7	92.9	89.5
Black/African American	4.2	3.3	4.3
Hispanic	0.1	1.9	0.2
Native American	3.3	0.0	3.5
Asian	2.7	1.9	2.5

Note.

a In this data set, child sex was conceptualized as a binary variable (i.e., male/female).

**Table 2.** Descriptive and correlation

	1	2	3	4	5	6	7	8	9	10	11	12	13	14	15	16	17	18	19
1. CP W1																			
2. PP W1	−.15																		
3. NP W1	.13	−.64																	
4. Antisocial W1	.16	−.28	.16																
5. CP W2	.50	−.12	.10	.14															
6. PP W2	−.25	.58	−.40	−.09	−.19														
7. NP W2	.20	−.40	.56	.08	.41	−.61													
8. Antisocial W2	.14	−.25	.13	.48	.11	−.12	.12												
9. CP W3	.36	.20	.09	.27	.51	−.21	.27	.26											
10. PP W3	−.17	.31	−.19	−.02	−.13	.38	−.25	−.14	−.19										
11. NP W3	.32	−.18	.12	.21	.32	−.30	.22	.19	.30	−.16									
12. Antisocial W3	.13	−.14	.12	.47	.19	−.14	.10	.62	.20	−.04	.18								
13. CP W4	.40	−.14	.11	.25	.51	−.13	.11	.16	.52	−.13	.25	.26							
14. PP W4	−.16	.39	−.27	−.05	−.14	.32	−.19	−.10	−.20	.61	−.12	−.08	−.22						
15. NP w4	.25	−.24	.10	.19	.33	−.18	.10	.20	.29	−.12	.61	.25	.42	−.23					
16. Antisocial W4	.15	−.19	.09	.34	.08	−.06	.05	.50	.19	−.06	.22	.55	.28	−.09	.23				
17. Alcohol W5	.10	−.05	.02	.11	.10	−.08	.04	.04	.19	.06	.07	.05	.32	.01	.08	.10			
18. Marijuana W5	.08	.06	.01	.09	.06	−.09	.12	.13	.17	−.08	.07	.13	.20	−.11	.08	.08	.44		
19. Cigarette W5	.11	.03	.03	.10	−.05	−.07	.06	.10	.20	−.08	.08	.10	.38	−.09	.15	.09	.41	.53	
*M (SD)*	.15 (.15)	5.79 (.48)	2.97 (.41)	9.43 (6.61)	.15 (.15)	5.81 (.44)	2.97 (.37)	3.12 (3.92)	.13 (.16)	3.22 (.41)	1.24 (.49)	3.29 (3.92)	.17 (.77)	3.13 (.47)	1.14 (.50)	2.39 (3.03)	1.68 (3.17)	.77 (1.61)	.56 (1.11)

*Note*. CP = conduct problems, W = wave, PP = positive parenting, NP = negative parenting.

**Table 3.** Random intercept cross-lagged panel model results

	*β* (SE)	95% CI
**Within-person effects**		
**Wave 1 -**> **Wave 2**		
Positive parenting W1 -> positive parenting W2	.366. (.090)***	.196, .514
Positive parenting W1 -> negative parenting W2	−.092 (.146)	−.379, .196
Positive parenting W1 -> CP 2	.175 (.108)	−055, .408
Positive parenting W1 -> parent antisocial W2	−.239 (.342)	−.406, .108
Negative parenting W1 -> positive parenting W2	−.034 (.097)	−.247, .160
Negative parenting W1 -> negative parenting W2	.380 (.090)***	.204, .557
Negative parenting W1 -> CP 2	.053 (.159)	−.176, .294
Negative parenting W1 -> parent antisocial W2	−.017 (.130)	−.413, .190
CP W1 -> positive parenting W2	−.061 (.089)	−.248, .113
CP W1 -> negative parenting W2	.196 (.062)*	.011, .361
CP W1 -> CP W2	.413 (.108)***	.201, .625
CP W1 -> parent antisocial W2	−.057 (.194)	−.438, .324
Parent antisocial W1 -> positive parenting W2	−.105 (.147)	−.391, .180
Parent antisocial W1 -> negative parenting W2	.033 (.171)	−.302, .368
Parent antisocial W1 -> CP W2	.150 (.114)	−.059, .380
Parent antisocial W1 -> parent antisocial W2	.686 (.212)***	.270, .989
**Wave 2 -**> **Wave 3**		
Positive parenting W2 -> positive parenting W3	.360 (.094)***	.137, .676
Positive parenting W2 -> negative parenting W3	−.013 (.079)	−.142, .167
Positive parenting W2 -> CP W3	−.086 (.146)	−.383, .221
Positive parenting W2 -> parent antisocial W3	−.144 (.152)	−.452, .144
Negative parenting W2 -> positive parenting W3	.096 (.078)	−.057, .248
Negative parenting W2 -> negative parenting W3	.291 (.069)***	.056, .458
Negative parenting W2 -> CP3 W3	.211 (.103)*	.008, .403
Negative parenting W2 -> parent antisocial W3	−.002 (.163)	−.322, .318
CP W2 -> positive parenting W3	−.041 (.081)	−.198, .117
CP W2 -> negative parenting W3	.298 (.061)***	.172, .424
CP W2 -> CP W3	.329 (.061)***	.134, .649
CP W2 -> parent antisocial W3	.132 (.189)	−.051, .352
Parent antisocial W2 -> positive parenting W3	−.063 (.087)	−.234, .107
Parent antisocial W2 -> negative parenting W3	.175 (.077)*	−.005, .259
Parent antisocial W2 -> CP W3	.193 (.092)*	.009, .397
Parent antisocial W2 -> parent antisocial W3	.536 (.133)***	.274, .797
**Wave 3 -**> **Wave 4**		
Positive parenting W3 -> positive parenting W4	.451 (.164)***	.175, .695
Positive parenting W3 -> negative parenting W4	−.008 (.049)	−.103, .088
Positive parenting W3 -> CP W4	−.062 (.050)	−.174, .039
Positive parenting W3 -> parent antisocial W4	−.022 (.075)	−.168, .124
Negative parenting W3 -> positive parenting W4	−.006 (.075)	−.152, .141
Negative parenting W3 -> negative parenting W4	.424 (.125)***	.181, .669
Negative parenting W3 -> CP W4	.233 (.066)*	.030, .237
Negative parenting W3 ->parent antisocial W4	.092 (.078)	−.061, .244
CP W3 -> positive parenting W4	−.024 (.107)	−.234, .186
CP W3 -> negative parenting W4	.278 (.092)**	.133, .478
CP W3 -> CP W4	.360 (.097)***	.169, .551
CP W3 -> parent antisocial W4	.013 (.058)	−.125, .100
Parent antisocial W3 -> positive parenting W4	−.070 (.085)	−.290, .098
Parent antisocial W3 -> negative parenting W4	.136 (.076)	−.005, .295
Parent antisocial W3 -> CP W4	.211 (.087)*	.039, .382
Parent antisocial W3 -> parent antisocial W4	.516 (.134)***	.253 .780
**Wave 4** -> **Wave 5**		
Positive parenting W4 -> alcohol W5	.072 (.046)	−.019, .162
Positive parenting W4 -> Marijuana W5	−.084 (.051)	−.182, .015
Positive parenting W4 -> cigarettes W5	−.040 (.039)	−.136, .056
Negative parenting W4 -> alcohol W5	−.068 (.047)	−.159, .024
Negative parenting W4 -> Marijuana W5	−.084 (.053)	−.188, .020
Negative parenting W4 -> Cigarettes W5	.038 (.059)	−.078, .154
CP W4 -> alcohol W5	.399 (.064)***	.274, .525
CP W4 -> marijuana W5	.393 (.058)***	.279, .506
CP W4 -> cigarettes W5	.408 (.066)***	.290, .527
Parent antisocial W4 -> alcohol W5	.014 (.082)	−.147, .174
Parent antisocial W4 -> marijuana W5	.014 (.056)	−.095, .123
Parent antisocial W4 -> cigarettes W5	−.011 (.059)	−.127, .105
**Between-person effects**		
Positive parenting with		
Negative parenting	−.666 (.045)***	−.701, −.503
CP	−.560 (.033)***	−.625, −.494
Parent antisocial	−.486 (.050)***	−.575, −.394
Negative-parenting with		
CP	.382 (.044)***	.296, .468
Parent antisocial	.338 (.040)***	.261, .416
CP with		
Parent antisocial	.319 (.045)***	.232, .406

*Note*. CP = conduct problems, W = Wave.

* *p* < .05,

** *p* < .01,

*** *p* < .001.
