# Supplementary material for: Pathways to substance use: Examining conduct problems and parenting behaviors from preschool to adolescence
Source: Dev Psychopathol. Author manuscript; Available in PMC 2024 Aug 1. (PMC10404304; doi:10.1017/S0954579422001328)
Supplement: 1 [file NIHMS1851119-supplement-1.docx]

| Supplementary Table 1: N’s of available data and substance use reported | | | | | | | | | |  |
| --- | --- | --- | --- | --- | --- | --- | --- | --- | --- | --- |
|  | Child Age  Mean (SD) | Positive Parenting | Negative Parenting | Parent Antisocial | Conduct Problems | Alcohol | Marijuana | Cigarettes |  |  |
| Wave 1 | 4.40 (1.01) | 380 | 380 | 688 | 579 | NA | NA | NA |  |  |
| Wave 2 | 7.61  (.96) | 308 | 308 | 677 | 625 | NA | NA | NA |  |  |
| Wave 3 | 10.51 (.93) | 464 | 464 | 689 | 659 | NA | NA | NA |  |  |
| Wave 4 | 13.51 (.92) | 405 | 405 | 589 | 666 | NA | NA | NA |  |  |
| Wave 5 | 16.58 (.97) | NA | NA | NA | NA | 609 | 608 | 609 |  |  |
| Use reported  N (%) | NA | NA | NA | NA | NA | 281 (39.80%) | 179  (25.35%) | 185  (26.20%) |  |  |
| Note. NA = not applicable; Total sample N=706 | | | | | | | | | | |
